# Typology, network features and damage response in worldwide urban road systems

**DOI:** 10.1371/journal.pone.0264546

**Published:** 2022-03-01

**Authors:** Jesus Felix Bayta Valenzuela, Erika Fille Tupas Legara, Christopher Pineda Monterola

**Affiliations:** Asian Institute of Management, Aboitiz School of Innovation, Technology and Entrepreneurship, Makati City, Philippines; China University of Geosciences, CHINA

## Abstract

We survey the network properties and response to damage sustained of road networks of cities worldwide, using OpenStreetMap (OSM) data. We find that our primary damage response variable $\bar{t_{1.0}}$, which is the average shortest time needed to reach all nodes in a road network (which stand in for locations within a metropolitan area) from an initial node (which stands in for the location of a center for disaster relief operations), is strongly linearly–correlated with *p*_*d*_, the fraction of the road network segments damaged. We find that this result, previously reported for a city’s road network as opposed to grid and scale-free idealizations, is widely present across the road networks we have examined regardless of location. Furthermore, we identify three families of road networks according to their damage response, forming a typology by which we can classify city road networks. Using this typology, we identify the family of road networks which may be of most concern from a humanitarian standpoint. We also find that, of the properties of the road networks we examined, the average shortest path length, 〈*l*_*min*_〉 and the average node degree, 〈*k*〉, proxies for city road network size and complexity respectively, are very significantly–correlated with damage susceptibility. In addition to forming a damage response typology by which city road networks could be classified, we consider five cities in detail, looking at risks and previous disaster events. Our results offer a generalizable framework in evaluating the feasibility of coursing relief efforts within disaster–affected areas using land–based transportation methods. They also provide, albeit in retrospect, a glimpse of the time difficulties which occurred, and the stakes of life involved in the humanitarian crisis which developed in the Kathmandu area due to the earthquakes of April and May 2015.

## Introduction

The death toll and property losses from natural disasters number in the thousands of lives and millions in US dollars annually. In 2018 alone, there were 315 natural disasters reported by the Centre for Research on the Epidemiology of Disasters (CRED), affecting 68.5 million people, causing 11,804 deaths, and US$ 132 billion in economic losses. While lower than the annual averages over the previous decades, the losses are still significant in a year dominated by storms and flooding [1].

Natural disasters, and disaster events in general, cause massive loss of life and property when they occur, losses which mount in the aftermath unless relief measures are promptly set in motion, and much—needed supplies reach those affected. The need for quick action and transportation of supplies is well—recognized among those involved in such efforts; the existence of a time window is commonly—held, ranging from 36 [2] to 72 hours [3, 4] for search, rescue and emergency relief efforts to take effect in the immediate aftermath of a disaster before death tolls rise (and after which emergency response efforts shift to restoration and recovery, and are no longer considered immediate). Without substantial commitment of aerial assets (and/or riparian and marine assets when the disaster location is sufficiently—close to rivers or the sea), such relief efforts necessarily have to make do with the existing land—based road networks. Even with the commitment of such capabilities, the road network infrastructure will still be needed, to a degree, for transportation of relief goods and supplies. Previous results [5, 6] indicate that idealizations of road networks to standard network types like grids or power law distributions result in significant variations in computed parameters for logistical operations during disasters. Hence, there is a need to incorporate the empirical distribution of an actual network, including its capacity during an emergency situation.

Relief efforts for a metropolitan area affected by a disaster presents a particularly—important case. A significant fraction of a nation’s population, wealth, and creative forces is typically concentrated in its urban areas. In 2018, 4.2 billion of the global population (55%) resided in urban settlements [7], a proportion which is projected to reach 68% by 2050—nearly the reverse of what it had been in 1950. Also in 2014, the world’s 300 largest metropolitan areas accounted for 20% of its population and 47% of its economic output [8]. As such, disasters affecting such areas are likely to cause losses of life and wealth far more significant than what would otherwise be the case. While many of these metropolitan areas (including many of the world’s capital cities and conurbations), for historical reasons, are situated by rivers or seas which may facilitate the delivery of emergency supplies, such rivers and seas are also potential sources of disaster events: either hydrological (flooding), meteorological (cyclone landfalls) or seismic (tsunamis) in origin. An additional source of concern is that many cities worldwide, for similar historical reasons, grew up in, or near to, geologically—active regions, which pose the risks of disasters stemming from volcanic eruptions and earthquakes. Major cities located on the rim of the Pacific Ocean are especially at risk, due to the rim coinciding for the most part with the Pacific Ring of Fire.

It may be expected that the road networks in urban locations may be more developed than those of the countryside, the rural areas, in the sense of the presence of more roads. However this may not suffice to ensure speedy transportation of relief supplies within the urban area spanned by its road network. It thus becomes necessary and natural to treat the road infrastructure from a network or graph perspective. Several characteristic quantities can be computed from a network representation of the road infrastructure, quantities which may contribute to the response and resilience of transportation using that road network to damage brought on by disaster. Network-theoretic perspectives have been used previously to probe road networks in general [9, 10] and specifically, questions of robustness, resilience and recovery in the face of disaster events [5, 11–24].

In a previous work [5], we examined the robustness of the capability of centrally-sourced relief operations to reach disaster-affected areas via roads from a network—theoretic perspective, with the city of Tacloban in the Philippines (hit by Typhoon Haiyan in November 2013), and two idealized networks of the same size (a scale—free network and a two—dimensional grid) serving as case studies. We found that $\bar{t_{q}}$, the average time to reach a fraction *q* of the nodes in the road network from a randomly—chosen starting node (serving as a relief center), increases linearly with the degree of damage the network sustained, *p*_*d*_ for Tacloban’s road network, in contrast with the two idealizations, under a variety of road damage scenarios. In this paper, we perform a similar analysis (robustness of $\bar{t_{q}}$ to road damage) for the road networks of cities around the world at two different times: 2014 (201 cities) and 2019 (194 cities). In addition, we characterize families of urban road networks according to their damage response, forming a typology by which we can classify such networks.

For each road network, we calculate several network properties, as well as its response to damage sustained by its road segments. Thus, we are able to identify which network properties significantly contribute to the ease (or difficulty) of channeling relief efforts through a road network. In addition, we identify key network characteristics of cities which may stand to lose the most, both in terms of lives and wealth, to delays in the relief effort in the aftermath of a disaster. Finally, we compare the network characteristics so identified between 2014 and 2019, in order to determine whether there have been shifts.

Our proposed typology of urban road networks thus complements others which rely more on spatial characteristics, such as the distribution of the shape factor of bounded city blocks [25] or road segment orientations [26]. For planners involved with disaster preparedness, response and resilience planning, identifying families of city road networks with similar damage response may aid in the formulation on the strategic level of disaster mitigation and response plans widely applicable within a given family of city road networks.

## Materials and methods

### Geospatial data

**Fig 1:**
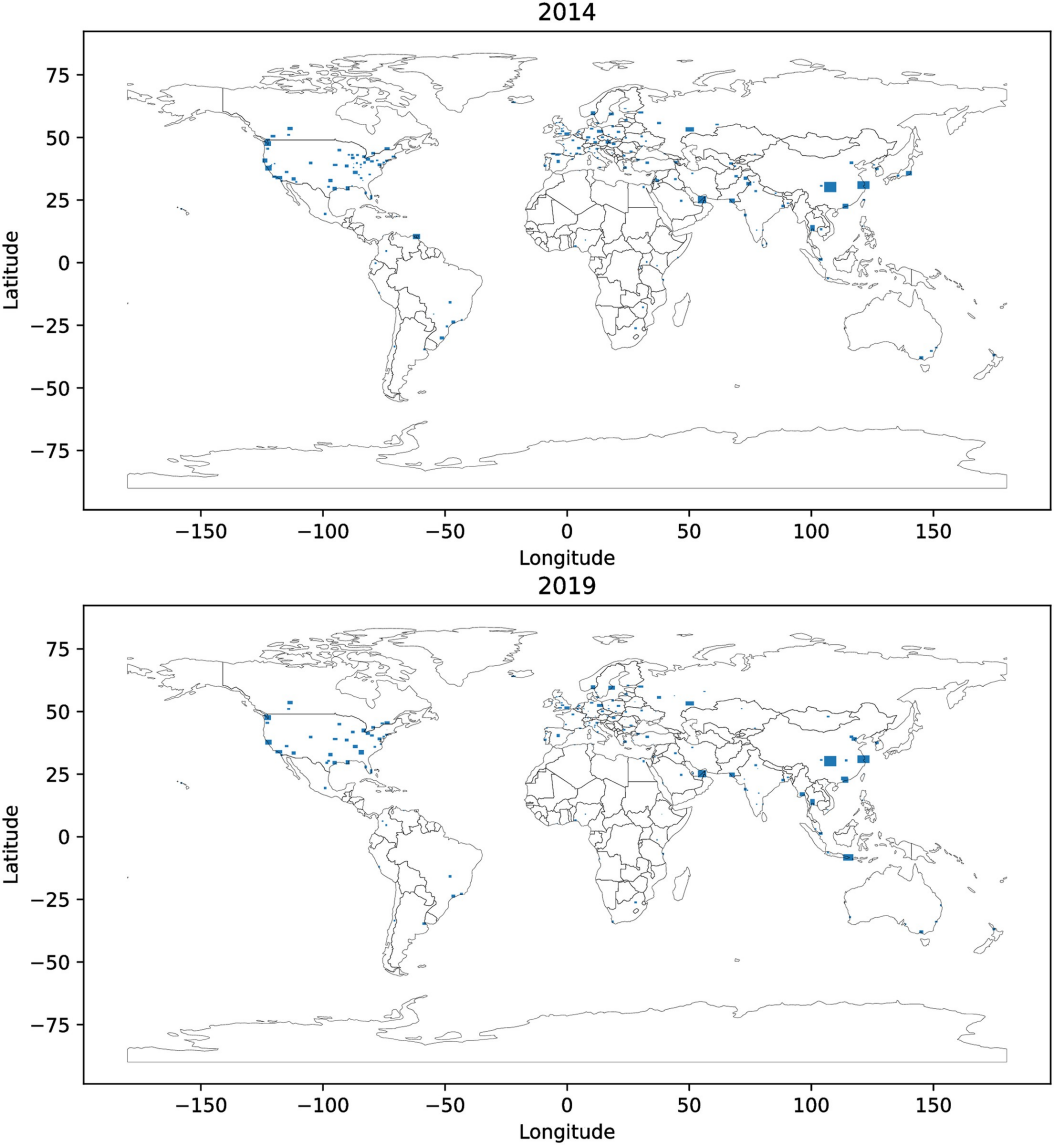
World map showing locations of downloaded city extracts for 2014 and 2019. The geodata used to render the plot is from OpenStreetMap [27] by way of Mapzen’s MetroExtracts (2014, [28]) and Nextzen’s MetroExtracts (2019, [30]) The figure was rendered using Python’s geopandas package [31].

2014 and 2018 map data for cities around the world were downloaded from OpenStreetmap (OSM) snapshots. OpenStreetMap [27] is the largest existing open and user-driven geospatial project covering the entire world. Extracts (subsets of OSM data covering smaller areas) for the cities were downloaded from two Metro Extracts websites: 2014-vintage extracts for 201 cities through Mapzen [28] (which shut down in February 2018 [29]), and 2019-vintage extracts for 194 cities through Nextzen [30], which provides the same framework as Mapzen. In both cases, all city extracts available at the time of data download (2014 for the Mapzen extracts and 2019 for the Nextzen extracts) were downloaded. Fig 1 shows the locations of the city extracts for both datasets (2014 and 2019).

**Fig 2:**
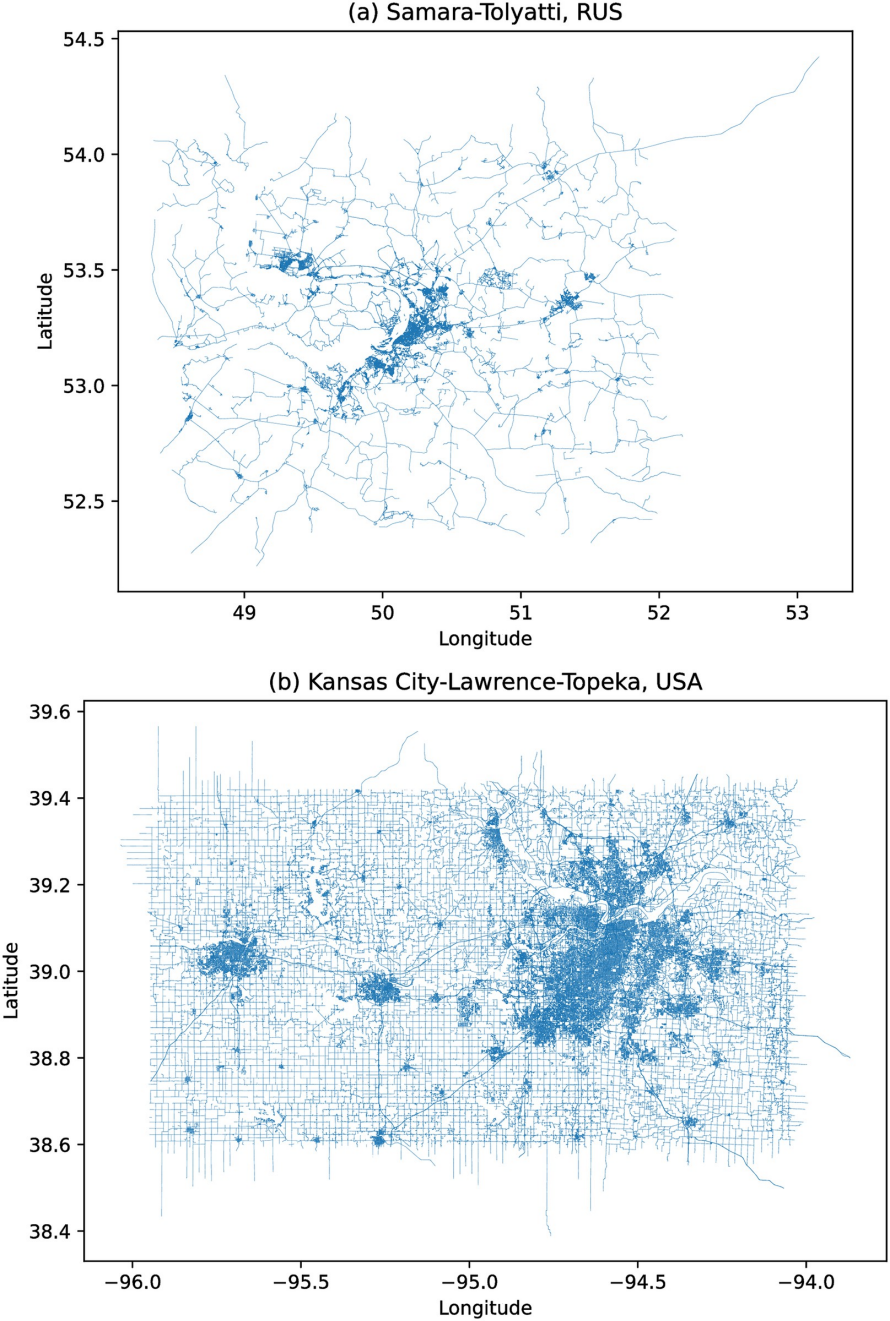
Conurbations of Samara-Tolyatti (Russia) and Kansas City-Lawrence-Topeka (USA) showing their road networks. The geodata used to render the plot is from OpenStreetMap [27] by way of Mapzen’s MetroExtracts [28]. The road networks were rendered using Python’s pyrosm package [32].

The data sets both contain several maps which refer to adjoining city units, such as the map data for the Samara and Tolyatti agglomeration in Russia and Kansas City, Lawrence and Topeka in the United States, while others, such as the data for the San Francisco Bay Area, refer to the extended urban agglomeration containing a central city (San Francisco in this case), whose data is also separately present in the datasets. Fig 2 shows the map datasets obtained from the first two urban areas mentioned.

The data contained in each map is organized as *nodes*, which represent points on the map, with latitude and longitude coordinates, *ways*, which connect any two nodes together, and *relations*, which group nodes and ways into map components, such as highways, buildings and other points of interest. From each map we extract the road networks by taking only those ways that are tagged as “highway” (which in OSM refers to any road [33], which may be tagged as anything from a trail to a trunk highway).

Demographic data was taken from the 11th and 15th editions of the survey of urban areas around the world collated and published by Demographia [34, 35]: specifically, the cities’ urban area in square kilometers, estimates of current population (based on projections by the United Nations agencies), and estimates of population density rounded to the nearest hundred persons per square kilometer. The survey took a city’s urban area as equal to its built—up area, and excluded rural land that otherwise fall within its administrative jurisdiction; on the other hand the built—up area may extend beyond the city’s formal bounds, such as the case of the city of Manila in the Philippines, whose built—up area not only covers its associated conurbation (the “National Capital Region”) but also extends substantially north, south and east into the latter’s adjoining administrative units. Out of the 201 metropolitan areas forming our 2014 OSM dataset, 185 have demographic data from the 2014 survey; Of the 194 in the 2019 OSM dataset, 188 have corresponding 2019 data.

Per-capita gross domestic product (GDP) for 2014 was taken from the report published by the Brookings Institution [8], containing the 300 cities around the world with the highest GDP per capita, adjusted according to purchasing power parity (PPP), which facilitates comparison across cities. Of the 201 areas in the 2014 OSM dataset, 130 are present in this report. More recent per-capita GDP estimates for cities are unavailable; however, the 2018 Brookings Institution report [36] presents GDP growth rates over two years, from 2014 to 2016.

In the following sections, the procedures we describe were applied to each dataset (2014 and 2019), unless stated otherwise.

### Road network damage response

Each road segment in OSM has a tag denoting its road type, which determines the speed at which vehicles are to traverse it and the time needed to do so. Following our previous work [5], we assign characteristic speeds for each road type to be able to estimate the time needed to traverse road segments of that type.

**Fig 3:**
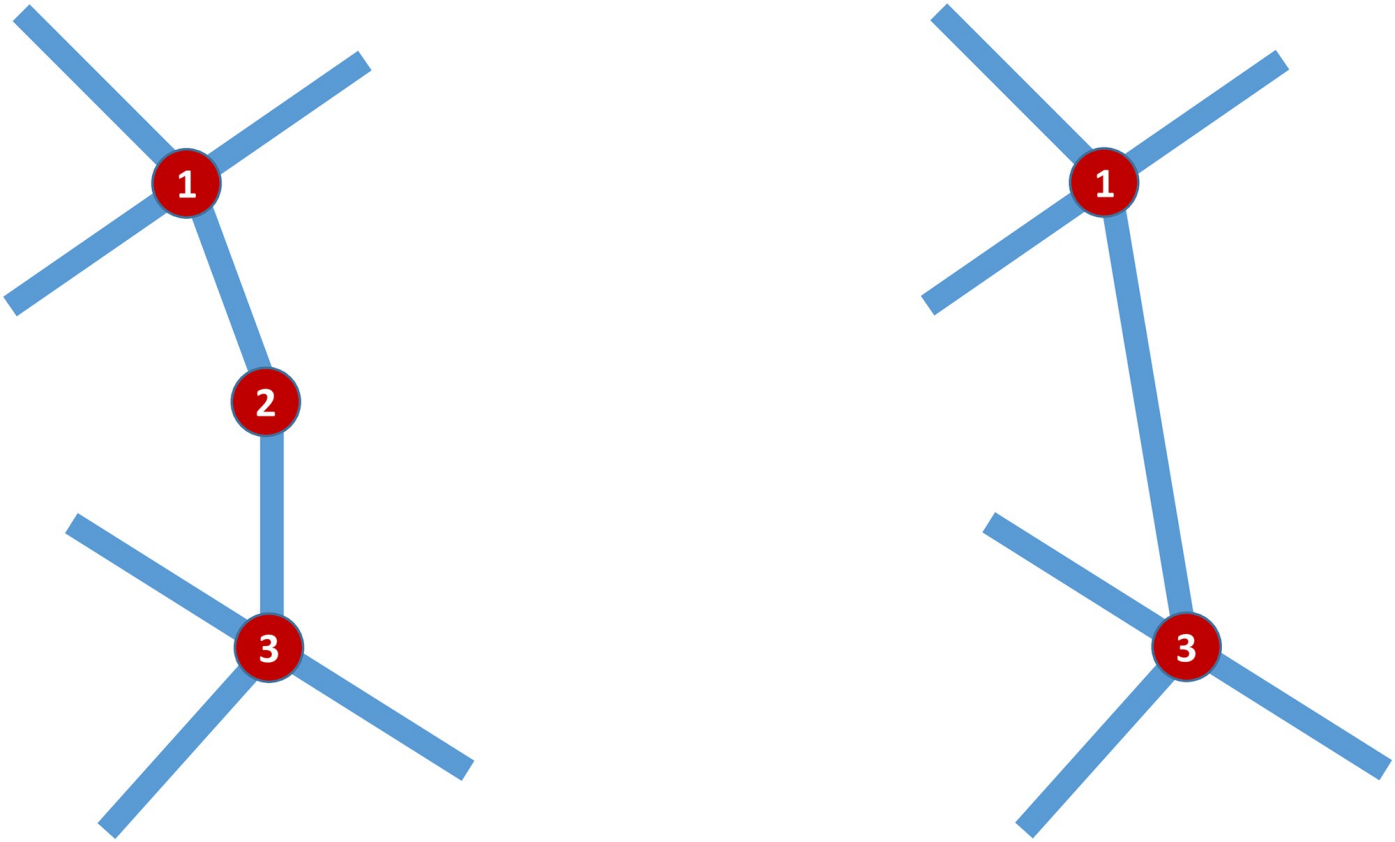
Schematic of a road network before (left) and after (right) application of the reduction process.

The road networks thus extracted have a substantial fraction of their nodes connected to only two others, representing two connected segments of a single road. As we want to examine the properties of the graph underlying this road network, we remove those nodes; thus, the nodes that are left in the graph represent the intersections present in the original road network. Fig 3 shows a schematic diagram of the process. We then obtain several network properties of each road network, using the approximate times to travel between nodes as edge weights. These are:

- The number of nodes, *N*;
- The number of edges, *E*;
- The average degree, 〈*k*〉;
- The network density, *D*;
- The average shortest path length, 〈*l*_*min*_〉, measured in time units;
- The average global clustering coefficient, 〈*C*_*global*_〉; and
- The average local clustering coefficient, 〈*C*_*local*_〉

These properties serve as the feature variables of the dataset.

To determine the damage response of the road networks, we determine $\bar{t_{q}}$, the average shortest time needed to reach a given proportion, *q*, of the nodes of a network from a randomly—chosen initial starting node, when a certain fraction *p*_*d*_ of road segments (also randomly—chosen) have sustained damage. The damage to a road segment is modeled as a reduction of 95% in the segment’s characteristic speed, and may represent the presence of debris littering the segment, or else structural damage. We find that the resulting damage response is approximately linear across all the cities we examined this way, and so we then obtain the slope, intercept and the square of the Pearson correlation coefficient of $\bar{t_{q}}$ against the *p*_*d*_ for *q* = 0.2, 0.4, 0.6, 0.8, and 1.0 for each road network. Here, the slope represents how sensitive the needed travel time is to the damage the network has sustained, and can thus be treated as the measure of susceptibility of the road network to damage. The intercept is the travel time in the absence of damage, while *r*^2^ can be treated as a measure of the predictability of the network’s damage response as given by the trend line.

**Fig 4:**
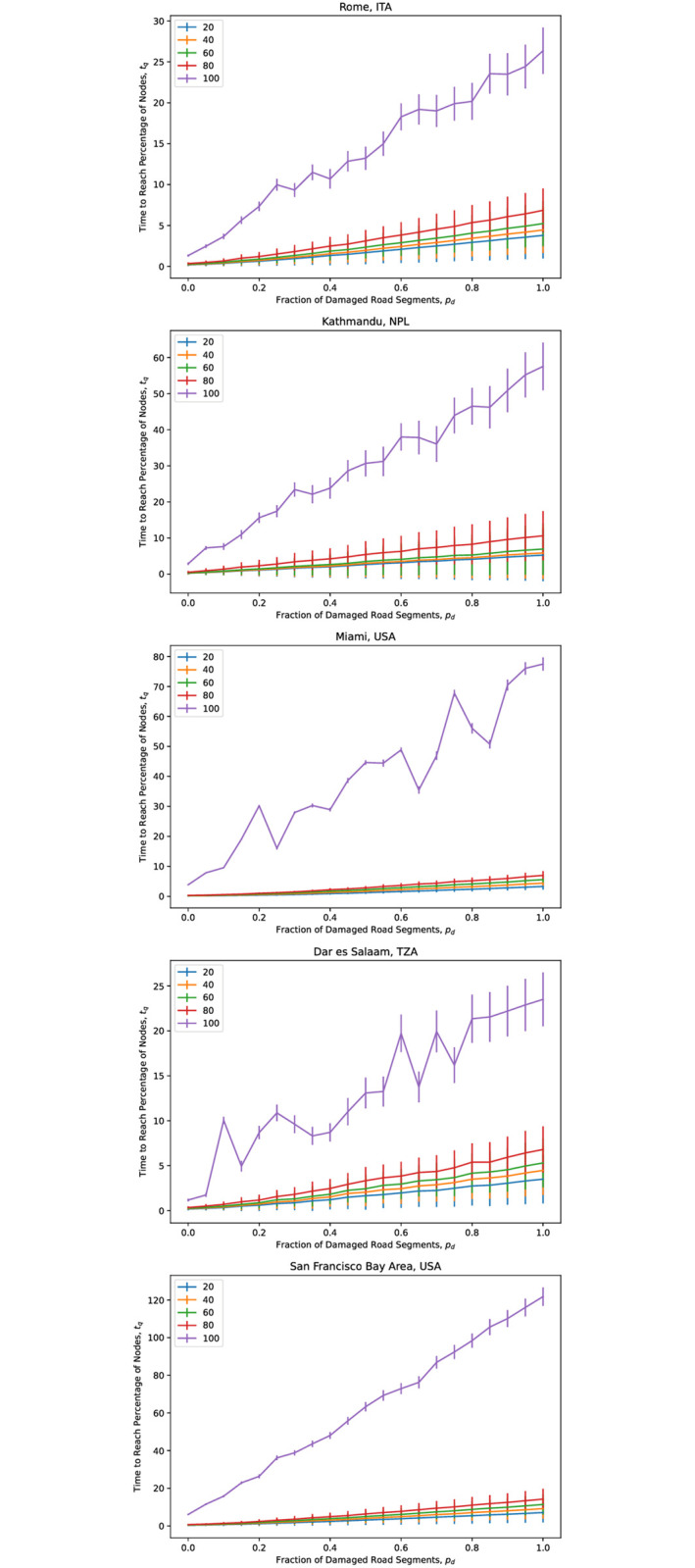
Dependence of $\bar{t_{q}}$, the time needed to reach a percentage *q* of nodes, on *p*_*d*_, the fraction of road segments damaged, for five sample city road networks. The road segments damaged were chosen at random. Averages were computed for 100 randomly—chosen trial locations for a relief center.

Across the road networks the results for *q* = 0.2, 0.4.0.6 and 0.8 are close to each other; this has significant implications further on. Fig 4 illustrates this result for five cities chosen without loss of generality and road networks taken from the 2014 dataset: Rome in Italy, Kathmandu in Nepal, Dar es Salaam in Tanzania, and from the United States, Miami and the San Francisco Bay Area.

Thus, we only take the slope, y—intercept and correlation coefficient for the *q* = 1.0 case, which is the average time needed to go from an initial starting node to all others by the shortest path. Incidentally, it can be shown that, under a mean—field approximation, this quantity, $\bar{t_{1.0}}$, is exactly the reciprocal of the average closeness centrality of the network, $\bar{C_{closeness}}$, and thus the latter should scale with $p_{d}^{-1}$.

### Clustering and model fitting

We obtain *Z*_*slope*_, *Z*_*intercept*_ and $Z_{r^{2}}$, the standardized values of each road network’s damage response variables—slope, intercept and *r*^2^ of $\bar{t_{1.0}}$ against *p*_*d*_, and do similarly for each road network’s feature variables (as enumerated previously). After standardization, we perform complete—linkage hierarchical clustering on the three damage response variables. The choice of the number of clusters for our purposes is a balance between exposing the desired amount of fine structure from the hierarchy of clusters, and the need for as few explanatory variables as possible (parsimony). We thus examine the hierarchical structure produced by the clustering algorithm using Tibshirani’s *gap statistic* [37].

**Fig 5:**
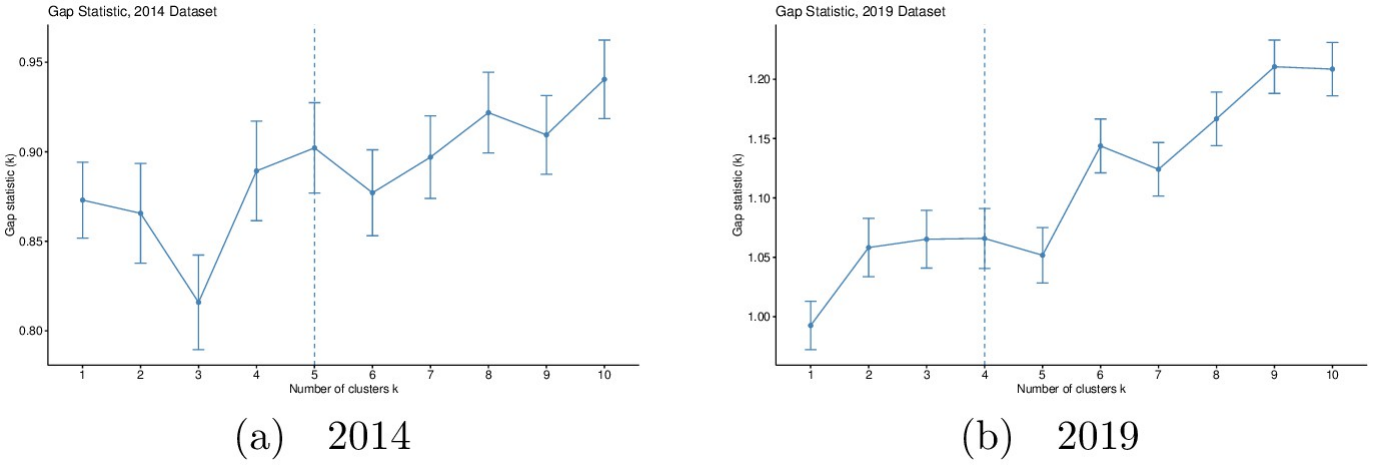
Gap statistic plots for standardized damage response variables (*Z*_*slope*_, *Z*_*intercept*_ and $Z_{r^{2}}$) of world cities in 2014 (a) and 2019 (b). The number of clusters, *k*, chosen for each dataset is marked by a dashed line. Clusters were generated using complete—linkage hierarchical clustering. The generation of clusters, gap statistics and plots were generated using implementations within R’s factoextra package.

For a choice of the number of clusters *k*, Tibshirani’s gap statistic, *Gap*_*n*_(*k*) is a measure of comparison between the intracluster dispersion obtained when the clustering algorithm is applied to the observed data, and that when the same algorithm is applied to a sample drawn from a null-hypothesis reference distribution (*e.g*. a spatially-uniform distribution). *Gap*_*n*_(*k*) is given by Eq 1:

$$\begin{array}{c} Gap_{n}(k)={\left\langle \text{log}(W_{k}^{*})\right\rangle }_{n}-\text{log}(W_{k}) \end{array}$$ (1)

where (*W*_*k*_) and $\left(W_{k}^{*}\right)$ are the intra-cluster dispersions (for *k* clusters) obtained from the observed data and a bootstrap sample generated from the null distribution, respectively, and 〈.〉_*n*_ means the average over a set of bootstrap samples, each of size *n*. Thus, for a given *k* a high value of *Gap*_*n*_(*k*) indicates the presence of *k* well-separated clusters. Fig 5 shows gap statistic plots for the damage response variables (*Z*_*slope*_, *Z*_*intercept*_ and $Z_{r^{2}}$) of 2014 and 2019 city map datasets.

Parsimony is commonly-enforced using the following heuristic: the minimum value of *k* is chosen such that *Gap*(*k*) ≥ *Gap*(*k* + 1) − *s*_*k*+1_, where *s*_*k*+1_ is the standard deviation (taken from the boostrapped samples) of *Gap*(*k* + 1). However, motivated by the need to expose more fine structure while still keeping the number of clusters low, we also look into the values of *Gap*(*k*). This leads us to choose *k* = 5 (2014) and *k* = 4 (2019) instead of *k* = 1 and *k* = 2 expected when we consider parsimony alone.

We then obtain a projection of the road networks’ standardized damage response using principal component analysis (PCA). The cumulative variance of first two principal components is approximately 0.96; thus two dimensions are sufficient for the projection. Table 1 shows the principal component loadings and cumulative variance of the three standardized damage response variables.

**Table 1:** Principal component loadings and cumulative variance of the standardized damage response of city road networks: Slope (*Z*_*slope*_), intercept (*Z*_*intercept*_) and *r*^2^ ($Z_{r^{2}}$). In both sets of city road networks, the damage response can effectively be reduced from three to two dimensions. The first component simultaneously encodes a road network’s susceptibility to damage; the second component, smaller in contribution than the first, encodes the unpredictability of the network’s damage response.

	*PC*_1_		*PC*_2_		*PC*_3_	
	2014	2019	2014	2019	2014	2019
*Z*_*slope*_	0.671	0.681	-0.353	-0.272	0.652	-0.680
*Z*_*intercept*_	0.718	0.707	0.091	0.002	-0.690	0.707
$Z_{r^{2}}$	-0.184	-0.191	-0.931	-0.962	-0.315	0.193
Cumulative variance	0.604	0.622	0.960	0.956	1.000	1.000

The component loadings for *PC*_1_ and *PC*_2_ are consistent across year, which allows us to use a common interpretation for each. The component loadings of the slope and intercept for *PC*_1_ have the same sign, while *r*^2^ has a smaller loading, with a sign opposite those of the slope and intercept. *PC*_1_ simultaneously encodes the damage response of a road network and its base “effective radius”; broadly speaking, it can be thought of as the *susceptibility* of a road network. *PC*_2_ has its highest loading from *r*^2^, along with a negative sign and minor contributions from the remaining two variables; as *r*^2^ encodes the closeness of the damage response to a linear fit, and thus the independence of $\bar{t_{1.0}}$ from the starting location on the road network, *PC*_2_ then encodes the location-*dependence* of $\bar{t_{1.0}}$. A higher value of *PC*_2_ implies greater variations in the damage response, and thus it can be thought of as the *unpredictability* of the road network’s damage response behavior.

We then fit a multiple linear regression model, separately for each combination of principal component (*PC*_1_ and *PC*_2_) and year (2014 and 2019), using the seven standardized network properties as predictor variables. Doing so allows us to run tests for each regression coefficient obtained, and thus to identify which network property has a significant association with a road network’s damage susceptibility (*PC*_1_) and unpredictability of damage response behavior (*PC*_2_).

## Results

### Road network response

Table 2 includes the mean and standard deviation of the quantities we computed in this work, including those of the response variables (slope, intercept and *r*^2^) for both 2014 and 2019 road network datasets. It is interesting to note that the average time to reach all locations on a road network from a starting point, $\bar{t_{1.0}}$ varies linearly with the fraction of the road segments damaged, *p*_*d*_ regardless of the location of the city, with *r*^2^ values all very high (≥0.98 on average). For the 2014 dataset, 0.985 is the average, with Campo Grande in Brazil being the city with the lowest value at 0.838. For the 2019 dataset, 0.990 is the average *r*^2^, with the Egyptian capital Cairo having the lowest, at 0.826.

**Table 2:** Mean and standard deviation for the computed network properties and damage response variables (slope, intercept and *r*^2^) for the road networks of 201 (2014) and 194 (2019) cities worldwide. Data extracted from OpenStreetMap (OSM) through the Metro Extracts services of Mapzen (2014) and Nextzen (2019).

Metric	Mean		Standard Deviation	
	2014	2019	2014	2019
〈*l*_*min*_〉	280.695	499.534	236.648	282.841
〈*C*_*global*_〉	9.790 × 10^−3^	1.809 × 10^−3^	6.921 × 10^−3^	1.278 × 10^−3^
〈*C*_*local*_〉	6.533 × 10^−3^	7.612 × 10^−4^	6.261 × 10^−3^	6.617 × 10^−4^
*D*	0.0104	2.481 × 10^−5^	0.0503	1.035 × 10^−4^
〈*k*〉	2.249	2.177	0.197	0.113
*N*	179015.1	368403.6	217516.8	388489.5
*E*	202236.6	397464.3	242111.2	413205.6
Slope	32.759	34.833	22.853	24.593
Intercept	1.689	1.698	1.390	1.382
*r* ^2^	0.986	0.990	0.020	0.017

**Fig 6:**
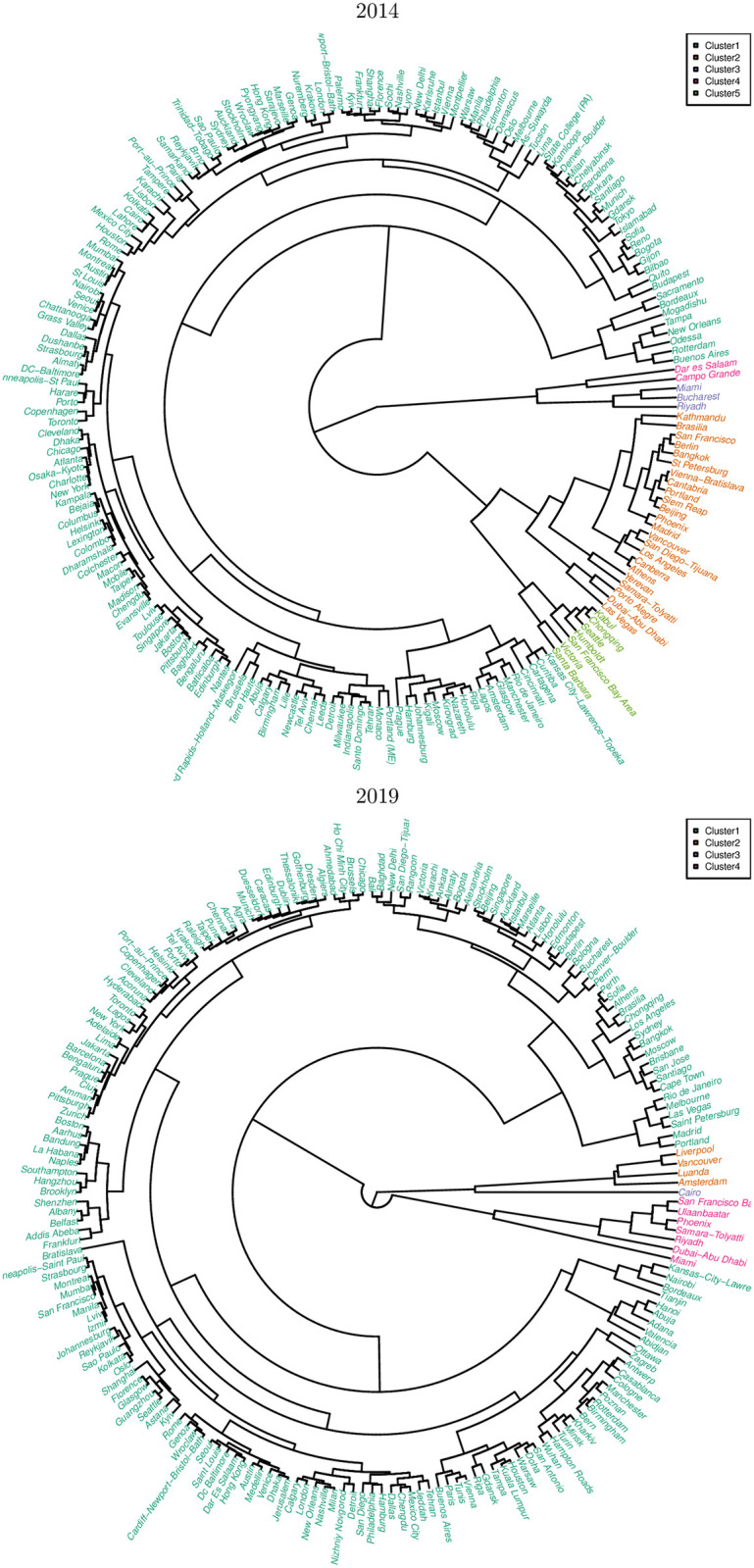
Road networks of 201 (2014) and 194 (2019) cities around the world. These are hierarchically clustered according to their standardized damage response variables: slope (*Z*_*slope*_), y—intercept (*Z*_*intercept*_) and *r*^2^ ($Z_{r^{2}}$). The number of clusters chosen for each set of road networks (5 for 2014, 4 for 2019) was selected taking parsimony and fine structure considerations into account, as discussed in the Methodology.

Fig 6 shows the dendrograms obtained from hierarchical clustering of the cities by distance in the three—dimensional space given by the standardized damage response variables *Z*_*slope*_, *Z*_*intercept*_ and $Z_{r^{2}}$. As discussed in the Methodology, we used Tibshirani *et al*.’s gap statistic to select particular values for the number of clusters: we selected five clusters for the 2014 road networks and four for the 2019 road networks, motivated by fine structure and parsimony considerations.

For the 2014 dataset, the union of Clusters 3 (Miami, Bucharest and Riyadh) and 4 (Dar es Salaam and Campo Grande) is the first to branch off, followed by the combined Clusters 2 (23 cities) and 5 (7 cities), which itself subsequently resolves into the two clusters. The remaining cities (166 in total) comprise Cluster 1, the largest cluster among the five in this dataset. For 2019, Cluster 2 (Liverpool, Vancouver, Luanda and Amsterdam) along with the singleton Cluster 3 (Cairo) are the first to branch off, followed by Cluster 4 (7 cities), with the vast majority under Cluster 1.

The consistently high *r*^2^ values we obtained for the damage response of city road networks for both datasets and shown above indicate that we have recovered a common, shared property of the road networks. In the following sections, we will ground our generalization of a typology of damage response upon this result.

### Damage response typology of urban road networks

The principal components of *Z*_*slope*_, *Z*_*intercept*_ and $Z_{r^{2}}$ are shown in Table 1. In both datasets (2014 and 2019), PCA yields two components accounting for 96% of the data variance, with *PC*_1_ encapsulating the contributions of the slope and the intercept and *PC*_2_ that of *r*^2^. The two variables with the strongest component loadings for *PC*_1_ (the linear dependence of the average time needed to reach all nodes in a road network from an initial starting point $\bar{t_{1.0}}$ on the fraction of the road network segments damaged *p*_*d*_ (the slope), and the average time to reach all nodes from an initial location in the absence of damage (the intercept)) are of most interest from the viewpoint of disaster relief operations, as they determine how fast any potentially—affected locations can be reached by relief efforts. The same sign of the coefficients of the two variables in *PC*_1_ also confirm the properties of the clusters we obtained, in which road networks with low (or high) *Z*_*slope*_ will also have low (or high) *Z*_*intercept*_. *PC*_2_, for its part, mostly encapsulates deviations from the linear trend as defined by the slope and the intercept and thus a measure of the *unpredictability* of the road network’s damage response, as observed previously.

In terms of damage response characteristics, we find three families of city road networks common to both datasets. This is justified by the fact that both dendrograms (2014 and 2019) in Fig 6 can be cut into three groups at a higher level than the actual number of clusters found (5 for 2014, 4 for 2019). In terms of the clusters obtained in the previous section, the correspondences between 2014 and 2019 are as follows: Clusters 1 and 2 (2014) to Cluster 1 (2019), Cluster 5 (2014) to Cluster 4 (2019), and more loosely, the outlying Clusters 3 and 4 (2014) to the outlying Clusters 2 and 3 (2019).

**Fig 7:**
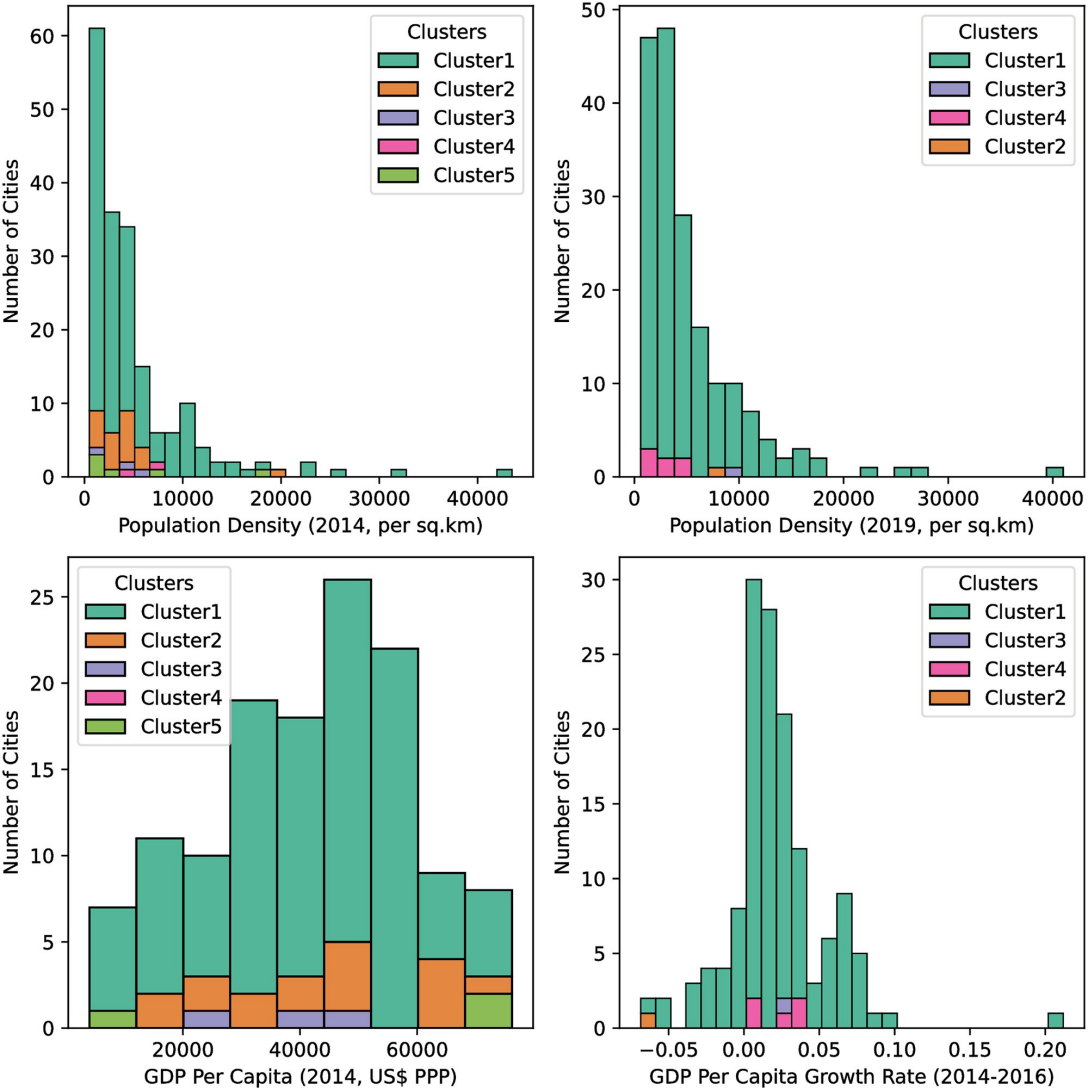
Frequencies for examined cities according to population density (2014 and 2019 datasets), 2014 GDP per capita (2014 dataset) and per-capita GDP growth from 2014 to 2016 (2019 dataset). For each road network dataset, the clusters obtained are colored as in their respective dendrograms in Fig 6.

Fig 7 shows how the cities in the two datasets are distributed according to population density (2014 and 2019 datasets), 2014 GDP per capita (2014 dataset) and per-capita GDP growth over 2014-2016 (2019 dataset), for those with obtainable data. We find no noticeable relationship between membership in the obtained clusters on one hand, and population density, per-capita GDP and GDP growth on the other, meaning that damage susceptibilities cut across population densities and wealth, and thus are of great concern to cities worldwide, wealthy or not, densely-populated or not.

The first family of cities, consisting of Cluster 1 (both 2014 and 2019) contains the majority of the city road networks, and are characterized by low *Z*_*slope*_, low *Z*_*intercept*_, and (generally) high $Z_{r^{2}}$, corresponding to low values for both *PC*_1_ and *PC*_2_. These span a wide range in both population density (as an example for the 2014 set Dhaka, Mumbai and Hong Kong are the densest urban areas while Mobile, Chattanooga and Macon, all in the United States of America, are the least dense) and GDP per capita (the American cities Boston, Houston and the Washington DC—Baltimore metropolitan area having the highest and Kolkata, Bengaluru and Chennai, all in India, having the lowest per-capita GDP in 2014 PPP-adjusted US dollars).

For these cities, points within their road networks need less time to be accessed in the absence of road damage (low *Z*_*intercept*_), and moreover, this ease of access is not very susceptible to increases in the damage (low *Z*_*slope*_); relief efforts can be conducted in them more easily in case of disaster events. This family of cities are of least concern, from the point of view of damage susceptibility.

The second family, consisting of Clusters 2 and 5 (2014) / Cluster 4 (2019), contains cities with both high *Z*_*slope*_ and *Z*_*intercept*_ (equivalently, high values of *PC*_1_), and low $Z_{r^{2}}$ (equivalently, low values of *PC*_2_). Similar to the first family, membership in this family is invariant of population density (with Canberra in Australia and Kathmandu in Nepal lying at the lower and upper ends in 2014) 2014 GDP per capita (Porto Alegre in Brazil and San Francisco in the United States), or 2014-2016 per-capita GDP growth (the Bay Area growing by 4.1% while Riyadh remained nearly-static at 0.2% over the same period). In contrast to the first family, this one contains cities within which locations are less readily—accessible even in the lack of disaster events, and become even less so with the increase in the level of damage the road network sustains. In the event of disasters affecting cities in this family, the use of their road networks to deliver relief within are bound to be highly—cumbersome and prone to delays, delays which workers can ill afford. Thus, these cities present the most concern.

**Fig 8:**
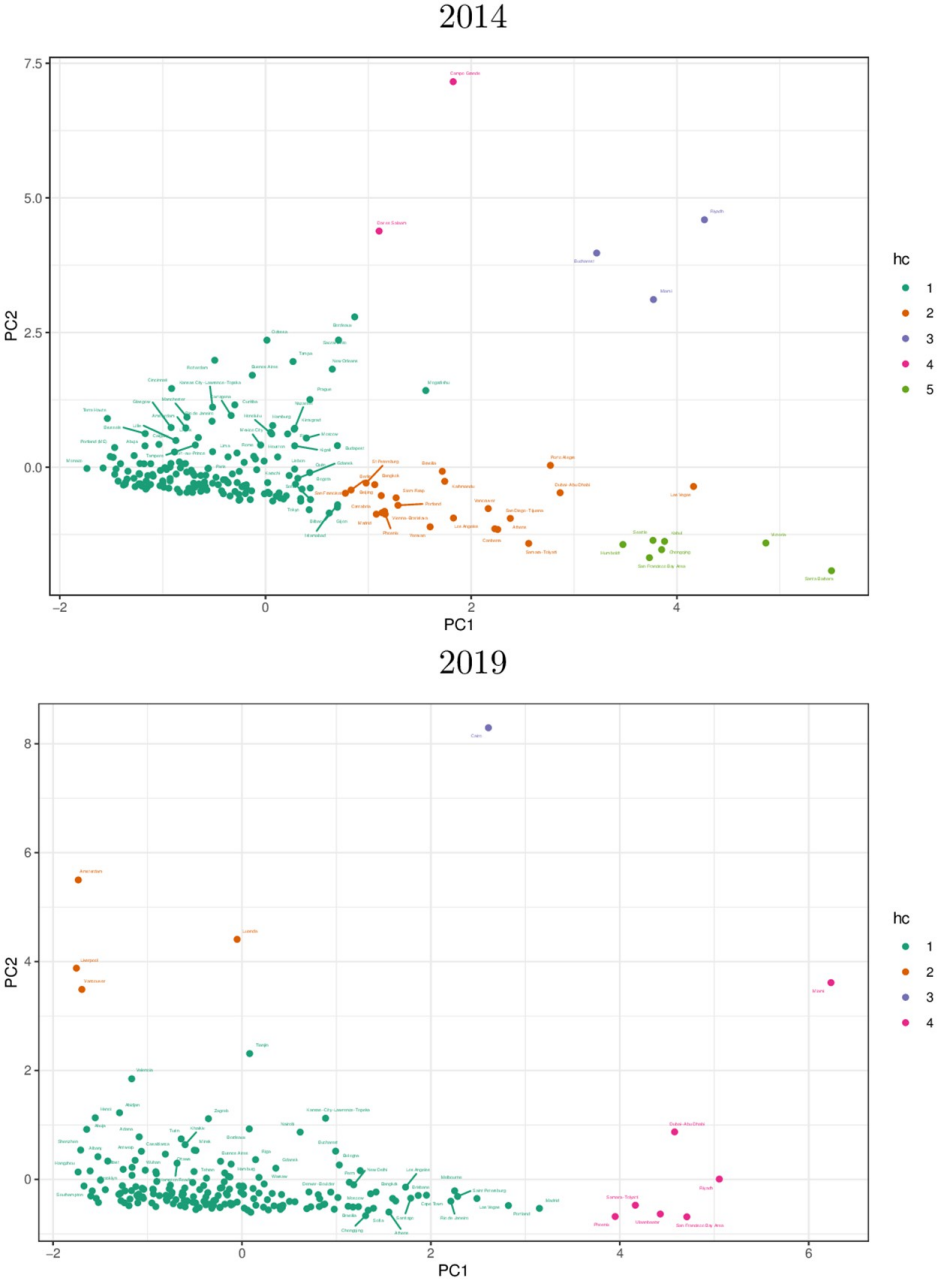
Principal-component projections of standardized damage response variables for the road networks of 201 (2014) and 194 (2019) cities worldwide: Slope (*Z*_*slope*_), intercept (*Z*_*intercept*_) and *r*^2^ ($Z_{r^{2}}$). For each road network dataset, the clusters obtained are colored as in their respective dendrograms in Fig 6.

The third family, consisting of the remaining clusters in both datasets, contains city road networks with very low $Z_{r^{2}}$ (high *PC*_2_), such as Campo Grande (2014) and Cairo (2019). These cities can collectively be considered as outliers which need more examination. The three families of city road networks can easily seen in Fig 8, which shows the projection of each city’s damage response variables (*Z*_*slope*_, *Z*_*intercept*_ and $Z_{r^{2}}$) onto the first two principal components obtained, *PC*_1_ and *PC*_2_. The cities of the first family have low *Z*_*slope*_ and *Z*_*intercept*_, thus clustering around the origin, while the cities of concern form a tail extending towards the bottom right, towards the region of high susceptibility to damage; the outliers are found elsewhere.

### Road network properties and damage response

**Fig 9:**
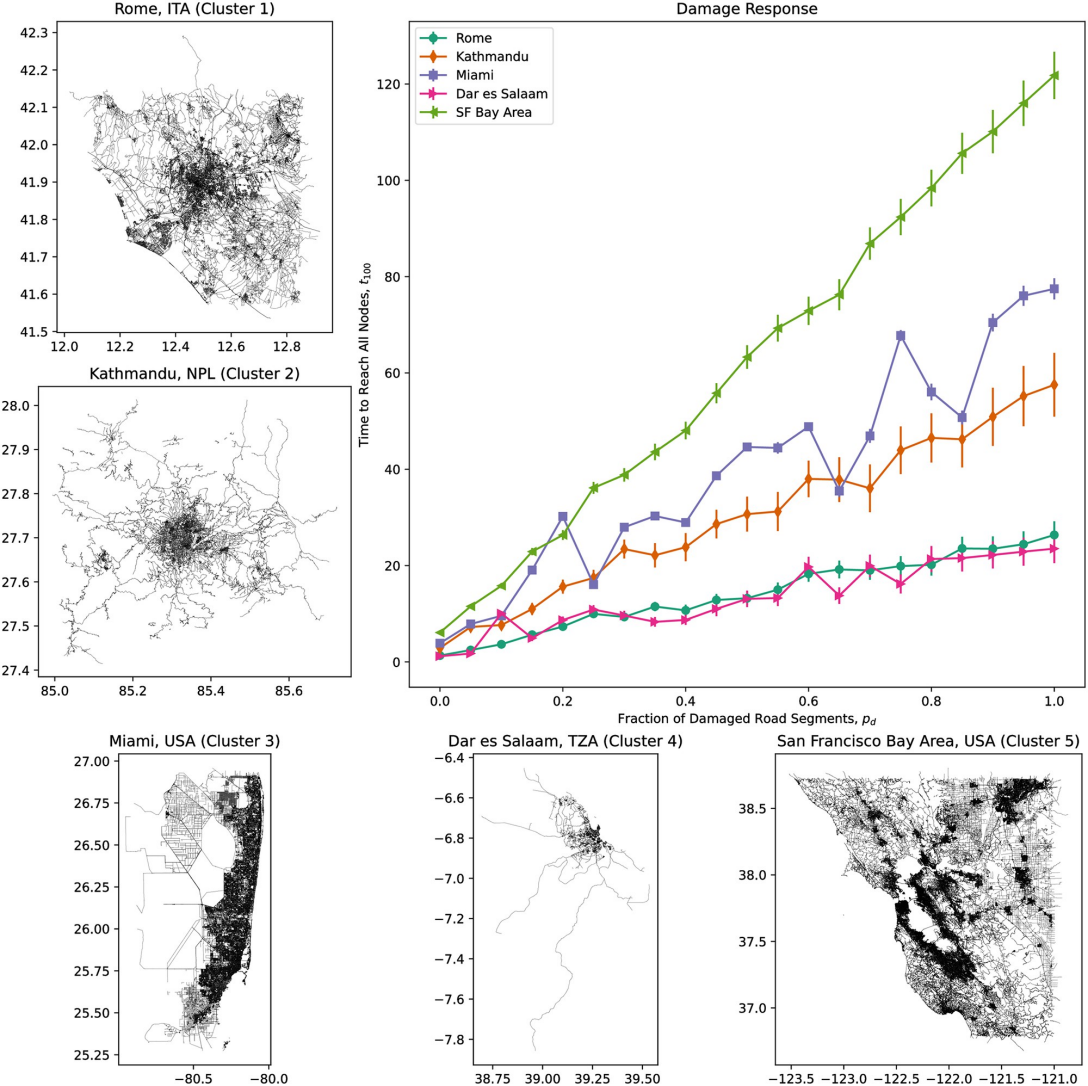
Damage response of five sample cities, together with their respective road networks. Each city and road network was selected from one 2014 cluster each.

Let us examine the damage response of the five sample cities previously mentioned, each belonging to a 2014 cluster: Rome (Cluster 1), Kathmandu (Cluster 2), Miami (Cluster 3), Dar es Salaam (Cluster 4) and the San Francisco Bay Area (Cluster 5). As seen previously in Fig 4, $\bar{t_{q}}$, the time to reach a *q* percentage of nodes in a network from an initial node representing a relief center, is only weakly dependent on the fraction of damaged road segments *p*_*d*_ for *q* up to around 80% to 90%. Thus, Fig 9 shows the time to reach the remaining, most inaccessible nodes (and thus, the entire network), *t*_100_ for the five cities, along with their respective road networks.

We see that the damage response of each city road network is approximately linear. Despite the response of Rome (Cluster 1 / “least concern”) and Dar es Salaam (Cluster 3 / “outlier”) tracking closely with each other, they are in different clusters, the small size / extent of Dar es Salaam compensates for the relative sparseness of its road network, in comparison with the bigger and more-connected network of Rome. Higher up are Kathmandu (Cluster 2, “of concern”) with its relatively sparse road network, and Miami (Cluster 3, “outlier”), whose road network is both larger and denser than that of Kathmandu, and have higher values for both susceptibility (*PC*_1_) and unpredictability (*PC*_2_). Finally, the road network with the steepest damage response (and thus, highest susceptibility) is that of the San Francisco Bay Area, which is also the biggest and densest road network of the five cities. This is no coincidence; as discussed below, a city road network’s size and complexity are the strongest significant predictors of its damage susceptibility.

We then fit multiple linear regression models to *PC*_1_ and *PC*_2_, using the standardized values of the seven computed network parameters as feature variables. The maximum likelihood estimates for the regression coefficients are shown in Table 3, along with the standard error and the result from a two—tailed *t*—test on each coefficient.

**Table 3:** Regression coefficients of generalized linear models for the principal components *PC*_1_ and *PC*_2_ for 2014 and 2019 city road networks. The standard error for each coefficient is in parentheses. Coefficients exhibiting statistical significance under a two-tailed *t*-test are marked with asterisks.

Feature	PC_1_		PC_2_	
	2014	2019	2014	2019
$Z_{\langle l_{min}\rangle }$	0.585(0.110)***	0.377(0.136)***	−0.251(0.080)***	−0.294(0.102)***
$Z_{\langle C_{global}\rangle }$	−0.079(0.312)	0.507(0.298)	−0.052(0.225)	−0.052(0.223)
$Z_{\langle C_{local}\rangle }$	−0.173(0.322)	−0.793(0.355)**	−0.180(0.232)	0.095(0.266)
*Z*_*D*_	0.210(0.124)	−0.063(0.104)	0.328(0.089)***	0.077(0.078)
*Z*_〈*k*〉_	0.513(0.174)***	0.390(0.197)**	0.791(0.126)***	0.041(0.147)
*Z*_*N*_	0.408(2.181)	−3.698(2.919)	4.299(1.573)***	0.928(2.189)
*Z*_*E*_	−0.565(2.168)	3.812(2.896)	−4.377(1.564)***	−0.892(2.172)
(intercept)	−0.000(0.088)	−0.000(0.092)	−0.000(0.063)	0.000(0.069)

** *p*<0.05

*** *p*<0.01

Among the coefficients of the regression models for *PC*_1_, those for $Z_{\langle l_{min}\rangle }$, associated with the average shortest path length are significant at *α* = 0.01 for both 2014 and 2019. The coefficient of *Z*_〈*k*〉_, the (standardized) average degree, is significant at *α* = 0.01 for 2014, but is significant at *α* = 0.05 for 2019. We fail to find statistically-significant associations between both clustering coefficients and *PC*_1_ for 2014; however we pick up a statistically-significant association between $Z_{\langle C_{local}\rangle }$ and *PC*_1_ at *α* = 0.05 for the 2019 dataset. Interestingly, the strength of the associations of $Z_{\langle l_{min}\rangle }$ and *Z*_〈*k*〉_, while remaining statistically significant at varying extents between 2014 and 2019, both drop, from 0.585 and 0.513 to 0.377 and 0.390, respectively, paralleled by the appearance of a strong negative (-0.793) and statistically significant (at *α* = 0.05) association between $Z_{\langle C_{local}\rangle }$ and *PC*_1_ in the 2019 road network dataset.

As *PC*_1_ encapsulates in the main the damage response properties of a road network in accordance with our clustering results, we thus identify 〈*l*_*min*_〉, 〈*k*〉, which are proxies for a city road network’s size and complexity, respectively, as the primary contributors to the susceptibility of a road network to damage: a road network with higher average path length and (to a slightly-lesser extent) higher average node degree will tend to require longer times to reach the most inaccessible locations within them, times which are moreover highly—dependent on the amount of damage the road network has sustained. For the 2019 dataset, 〈*C*_*local*_〉, the average local clustering coefficient, is a measure of the “small-worldness” of a network; high values imply ease of accessibility from one node in the road network to another; as well as more redundant connections and thus decreased susceptibility to damage, something borne out by the negative sign of its regression coefficient.

For *PC*_2_ and the 2014 dataset, we find five network properties with significant regression coefficients: 〈*l*_*min*_〉 (-0.251), *D* (0.328), 〈*k*〉 (0.791), *N* (4.299) and *E* (-4.377) Of these, the coefficients of $Z_{\langle l_{min}\rangle }$ and *Z*_*E*_ are both negative, indicating that their contributions have the effect of *lowering* *PC*_2_, and thus the unpredictability of the road’s damage response, while those of the other three serve to increase the latter. The 2019 dataset differs drastically from the 2014 one in this regard: only the coefficient of the average shortest path length (-0.294), remains statistically significant: 〈*l*_*min*_〉, and thus the network’s size, has a consistent negative contribution to damage response unpredictability (equivalently, a consistent positive contribution to the damage response *r*^2^, since the latter’s component loading for *PC*_2_ is *negative*) across datasets.

## Discussion

### Universality of small—Scale and large—Scale damage response

In a previous work [5], we reported that $\bar{t_{q}}$ increases linearly with the degree of damage the network sustained, *p*_*d*_ for a real road network, in contrast with two idealizations of a road network (a scale—free network and a two—dimensional grid). This implies that for a real road network, there is no equivalent to a percolation threshold for *p*_*d*_ which divides the response of $\bar{t_{q}}$ into two regimes (as was for the two idealizations). Furthermore, we reported that the time needed to reach lower percentages of the road network (*q* below 80% to 90%) has a weaker dependence on *p*_*d*_ (and thus exhibit stronger robustness to damage), in contrast to when *q* = 100, or equivalently, when the most inaccessible locations are also needed to be served. With this survey of two metropolitan road network datasets, we further find that these two insights are not unique to the city road network previously studied (Tacloban City in the Philippines, which was hit by Typhoon Haiyan in November 2013), but is also found in others worldwide. We conjecture that this is a universal property of the damage response of road networks, something which is supported by typological commonalities we found across the two road network datasets.

This combination of small-scale robustness combined with variable susceptibility at the greater scale of the entire city has important implications for disaster preparations: it means that for any city, there exists a minimum number of optimally—positioned relief centers that can robustly service places in its vicinity using the road network, such that the entire city can be robustly served by these centers when a disaster occurs. Any lower than this threshold and the existing centers will not be able to robustly—serve an entire city, with the most inaccessible places taking a much longer time to reach via the road network. Conversely, centers which are intended to serve an entire city’s extent will find the use of the road network impractical if they are to reach the farthest locations in a low enough time, and thus will have to use alternate means of service delivery (such as air or sealifts).

### Damage response and risks

By using hierarchical clustering of the (transformed) damage response variables of these road networks, we are ultimately able to classify cities into one of three types, according to the damage response characteristics of their road networks. The classification of cities is invariant of population density, GDP per capita or per-capita GDP growth, meaning that both low-and high-risk cities could be found at both extremes of each.

The five sample cities we have examined face a variety of risks: seismic (Rome, Kathmandu and the San Francisco Bay Area), hurricane (Miami) and floods (Dar es Salaam). Rome, belonging to the “least concern” family, is the most robust of the five, yet still has cause for concern, as it is near the Mt. Vettore fault in the Apennines, which in Classical and Late Antiquity caused substantial damage to its buildings and most recently strong earthquakes in 2016 [38]. The San Francisco Bay Area, which is of concern due to its large size / spatial extent, is near the San Andreas fault system, which is at risk for strong earthquakes and had caused the 1906 San Francisco earthquake and fire. Miami, on the east coast of the United Stats, faces a high risk of hurricanes, while Dar es Salaam perenially suffers from floods. Finally, Kathmandu lies within a seismically active region, where ongoing tectonic collision has led to the formation of the Himalayan belt. As mentioned below, this city, belonging to the “of concern” family in 2014, suffered massive loss of life and property when an earthquake struck in April 2015.

In addition, we have identified the relative contributions of various network properties to the damage response of road networks. Among these three families we have identified, it is the second type (characterized by both high base $\bar{t_{1.0}}$ and also high susceptibility of the latter to varying *p*_*d*_) which may offer the most concern, from the disaster relief viewpoint. Road—based relief efforts within the cities in this cluster are bound to be the most susceptible to the amount of damage the road network has sustained. Among the metropolitan areas of this type, the San Francisco Bay Area has the highest purchasing power—adjusted GDP per capita in 2014 ($75,382), followed by Seattle ($73,012), Portland ($67,639), Los Angeles ($65,082), and the cross—border San Diego and Tijuana ($62,295). Of these five areas, all except Portland lie on the seismically—active Pacific Ring of Fire, with the Bay Area, Los Angeles and San Diego—Tijuana in the vicinity of the San Andreas fault system; in case of a major earthquake these cities stand to sustain considerable impacts on life and wealth, and road—based relief efforts likely to suffer large delays. In such situations, alternatives to land—based relief delivery systems would be preferable. It must be said, however, that with per—capita GDP in these areas being high, the frameworks and infrastructure for these areas may be well—developed, and the actual impact due to a disaster event may be much less. Of the five mentioned metropolitan areas, only Seattle has moved out of this “of-concern” grouping as of 2019 data; per-capita GDP growth between 2014 and 2016 has also been positive except for San Diego area, which contracted by 0.4% over the same period [36].

In terms of the risk to populations, Kathmandu in Nepal had the highest population density among the cities of the second type in the 2014 dataset (19,800 persons per square kilometer) followed by Kabul (17,900), Chongqing (7,700), Athens (6,000) and Bangkok (5,800). Land—based relief efforts undertaken within these cities would suffer considerable delays in transportation. With the time window to reach affected populations being limited, more of the latter stand to lose if these cities are hit. Thus in April and May 2015, when earthquakes devastated Kathmandu and its environs, the relative remoteness of the area, compounded by the state of its roads, made transportation very difficult, and contributed to a grave humanitarian crisis. The 2019 dataset has the following cities of concern with the highest population densities: New Delhi (12,600 per square kilometer), Baghdad (10,900), Yangon (9,400), Sofia (6,400) and Rio de Janeiro (6,300), all of which are at risk from a variety of natural disasters such as earthquakes and flooding.

## Conclusion

In this work we have examined world urban areas at two different points in time (2014 and 2019), according to the response of their road networks to increasing amounts of damage. We show that a linear damage response behavior of a road network (as opposed to grid or scale-free idealizations of it) is widespread, and may be universal to city road networks. Thus, we sought, and are successful, in obtaining a typology of city road networks according to their damage response characteristics.

Using principal component analysis, we have identified two variables which characterize the damage response of city road networks: *susceptibility* and *unpredictability*. Thus, we are able to classify the road networks of cities we examined into three families. The majority of the city road networks belong to the first family, which exhibit low susceptibility (corresponding to both a low average time to reach all points on the network from a random starting point in the absence of damage, and a weak response to varying amounts of damage) and low unpredictability of damage response (equivalently, a high degree of consistency of the damage response to a linear pattern). These road networks thus are resilient to damage, and thus will facilitate quick deployment of relief efforts and distribution of emergency goods and services during times of disasters. The second family of city road networks, the vulnerable ones, is characterized by high damage susceptibility and low unpredictability, which makes this family of particular concern from a disaster response standpoint. For several cities in this family, having high population densities and already at risk from natural calamities such as earthquakes and flooding, high susceptibility of road networks to damage presents a complicating factor for emergency response and rescue efforts. The third, and smallest family of cities contains outliers, of which more information is needed.

Among the network properties we examined, we find that a city road network’s average shortest path length, 〈*l*_*min*_〉 and the average degree, 〈*k*〉 are significantly-associated with its damage susceptibility for both datasets we examined (2014 and 2019), while only 〈*l*_*min*_〉 has a significant association with damage response unpredictability across datasets. Thus, we contend that these two properties, which are proxies for a city’s size (〈*l*_*min*_〉) and the complexity of its road network (〈*k*〉) are the strongest predictors of a road network’s vulnerability to damage.

Thus, a network-based assessment of a city’s road infrastructure may offer valuable insights and identify systemic weaknesses—weaknesses which have to be taken into account when planning for emergency relief. For one city in particular, however, this report could only offer a post-mortem of sorts: Nepal’s capital Kathmandu had the highest population density among the vulnerable cities we identified in the 2014 road network dataset, something borne out by massive loss of life when the country was hit by an earthquake in early 2015.
